# The Mitochondrial Na^+^/Ca^2+^ Exchanger Inhibitor CGP37157 Preserves Muscle Structure and Function to Increase Lifespan and Healthspan in *Caenorhabditis elegans*

**DOI:** 10.3389/fphar.2021.695687

**Published:** 2021-06-15

**Authors:** Paloma García-Casas, Pilar Alvarez-Illera, Eva Gómez-Orte, Juan Cabello, Rosalba I. Fonteriz, Mayte Montero, Javier Alvarez

**Affiliations:** ^1^ Departamento de Bioquímica y Biología Molecular y Fisiología, Facultad de Medicina, Unidad de Excelencia Instituto de Biología y Genética Molecular (IBGM), Universidad de Valladolid and CSIC, Valladolid, Spain; ^2^ Center for Biomedical Research of La Rioja (CIBIR), Logroño, Spain

**Keywords:** *C. elegans*, CGP37157, lifespan, endoplasmic reticulum, mitochondria, sarcopenia

## Abstract

We have reported recently that the mitochondrial Na^+^/Ca^2+^ exchanger inhibitor CGP37157 extends lifespan in *Caenorhabditis elegans* by a mechanism involving mitochondria, the TOR pathway and the insulin/IGF1 pathway. Here we show that CGP37157 significantly improved the evolution with age of the sarcomeric regular structure, delaying development of sarcopenia in *C. elegans* body wall muscle and increasing the average and maximum speed of the worms. Similarly, CGP37157 favored the maintenance of a regular mitochondrial structure during aging. We have also investigated further the mechanism of the effect of CGP37157 by studying its effect in mutants of *aak-1;aak-2*/AMP-activated kinase, *sir-2.1*/sirtuin, *rsks-1*/S6 kinase and *daf-16*/FOXO. We found that this compound was still effective increasing lifespan in all these mutants, indicating that these pathways are not involved in the effect. We have then monitored pharynx cytosolic and mitochondrial Ca^2+^ signalling and our results suggest that CGP37157 is probably inhibiting not only the mitochondrial Na^+^/Ca^2+^ exchanger, but also Ca^2+^ entry through the plasma membrane. Finally, a transcriptomic study detected that CGP37157 induced changes in lipid metabolism enzymes and a four-fold increase in the expression of *ncx-*6, one of the *C. elegans* mitochondrial Na^+^/Ca^2+^ exchangers. In summary, CGP37157 increases both lifespan and healthspan by a mechanism involving changes in cytosolic and mitochondrial Ca^2+^ homeostasis. Thus, Ca^2+^ signalling could be a promising target to act on aging.

## Introduction

Mitochondria, the organelle responsible of aerobic energy production, plays also multiple roles in cellular Ca^2+^ homeostasis (Pizzo et al., 2012; Gherardi et al., 2020). Increase in mitochondrial [Ca^2+^] activates mitochondrial metabolism, linking cell activation to energy production. On the other hand, the rapid mitochondrial Ca^2+^-accumulation that occurs during cell stimulation constitutes a mechanism of transient buffering of cytosolic Ca^2+^ (Montero et al., 2000). This mechanism regulates a variety of phenomena triggered by cytosolic Ca^2+^, such as neurotransmitter secretion or muscle contraction. Moreover, mitochondria play also an important role in the regulation of cell death induced by mitochondrial Ca^2+^ overload.

The best known pathway for Ca^2+^ entry into the mitochondria is the mitochondrial Ca^2+^ uniporter (MCU), a Ca^2+^-activated Ca^2+^ channel present in the inner mitochondrial membrane (Gherardi et al., 2020), although additional pathways must be present to explain the persistence of mitochondrial Ca^2+^ signalling in MCU knockout models (Álvarez-Illera et al., 2020). Once cell stimulation is finished, cytosolic Ca^2+^ decreases, MCU becomes inactivated and Ca^2+^ is extruded from mitochondria through the mitochondrial Na^+^/Ca^2+^ exchanger (mNCX), the main Ca^2+^ exit pathway from mitochondria (Palty et al., 2010). Benzothiazepine CGP37157 is a potent inhibitor of mNCX. However, other targets with a similar dose-response relationship have also been reported. All of them involve the inhibition of other Ca^2+^ pathways, including plasma membrane L-type Ca^2+^ channels (Baron and Thayer, 1997), plasma membrane Na^+^/Ca^2+^ exchangers (Czyz and Kiedrowski, 2003), or CALHM1 Ca^2+^ channels (Moreno-Ortega et al., 2015).

We have reported recently that CGP37157 extends lifespan on *C. elegans* worms with a bell-shaped concentration–response, so that the effect was obtained at submaximal concentrations, with higher doses producing no effect (García-Casas et al., 2019). The actual target responsible for the lifespan effect in *C. elegans* is unknown, in part because the diversity of Na^+^/Ca^2+^ exchanger isoforms is much greater in *C. elegans*. Humans have only one isoform of mNCX, named NCLX (Palty et al., 2010). Instead, *C. elegans* has 10 different isoforms of Na^+^/Ca^2+^ exchangers (named *ncx-1* to *ncx-10*) and five of them are homologs of NCLX (*ncx-6* to *ncx-10*) with different tissue distribution (Sharma et al., 2013; He and O’Halloran, 2014; Sharma and O’Halloran, 2014). Out of the 10 *ncx* genes of *C. elegans*, only *ncx-9* has been studied in more detail, showing that it performs CGP37157-sensitive Na^+^/Ca^2+^ exchange activity in mitochondria (Sharma et al., 2017). In addition, *C. elegans* has also homologs of L-type Ca^2+^ channels (EGL-19) and CALHM1 Ca^2+^ channels (CLHM-1), the plasma membrane Ca^2+^ channels that are also sensitive to CGP37157 in mammalian cells, although it is not known whether the *C. elegans* homologs are sensitive to CGP37157 or not.

CGP37157 has also been shown to induce neuroprotection in several experimental models of neurotoxicity (Nicolau et al., 2009; Nicolau et al., 2010; González-Lafuente et al., 2012). Neuroprotection has been mainly attributed to inhibition of Ca^2+^ entry to neurons, either through voltage-dependent Ca^2+^ channels or through CALHM1 Ca^2+^ channels (Ruiz et al., 2014; Garrosa et al., 2020). Whether the mechanism of neuroprotection is related or not to the mechanism of lifespan increase is still unknown. Correlation between neuroprotection and lifespan extension has been reported before, e.g., the effect of partial mitochondrial uncoupling, which attenuates age-dependent neurodegeneration and increases survival in *C. elegans* (Lemire et al., 2009; Cho et al., 2017).

On the other hand, it is also important to determine if CGP37157 increases only lifespan, or if it is able to improve also healthspan, the healthy time of life. In some long-lived mutants, the increase in lifespan was achieved at the cost of an increase in frailty time (Bansal et al., 2015). We have therefore decided to monitor functional parameters such as speed of mobility and morphological structure of muscle sarcomere and mitochondria during aging, to test if this compound was able to improve the performance with respect to the controls. Our results show that CGP37157 increased mobility and prevented sarcopenia and mitochondrial degeneration with age.

Finally, as for the mechanism underlying the effects of CGP37157 on *C. elegans* lifespan, we have described that the effect disappeared in mutants of the mitochondrial respiratory chain (*nuo-6*), the TOR pathway (*daf-15*) and the insulin/IGF1 pathway (*daf-2*) (García-Casas et al., 2019), but further work was required to clarify the pathways involved. We have extended here this study by using mutants of *aak-1;aak-2*/AMP-activated kinase, *sir-2.1*/sirtuin, *rsks-1*/S6 kinase and *daf-16*/FOXO. In addition, we have performed measurements of the cytosolic and mitochondrial [Ca^2+^] oscillations in the presence and in the absence of this compound, and we have made a transcriptomic analysis to determine if CGP37157 induces changes in the expression of components of the pathways known to control lifespan, or in components of the Ca^2+^ signalling machinery. Our analysis reveals that CGP37157 acts downstream of *daf-16*/FOXO in the insulin pathway, involves TORC1 inhibition and functional mitochondria, induces changes in plasma membrane and mitochondrial Ca^2+^ fluxes, changes the expression of many lipid metabolism enzymes and increases the expression of the mNCX *C. elegans* homolog *ncx-6* gene.

## Materials and Methods

### *C*. *Elegans* Strains and Maintenance

Strains used were as follows: AQ2038, integrated strain expressing cytosolic yellow cameleon 2.1. (YC2.1) in pharynx controlled by the *myo-2* promoter (*pmyo-2::YC2.1*) (Alvarez-Illera et al., 2016), AQ2121, integrated strain expressing cytosolic yellow cameleon 2.1. (YC2.1) in body wall muscle cells controlled by the *myo-3* promoter (*pmyo-3::YC2.1*), both strains kindly provided by Drs. Robyn Branicky and W. Schafer, MRC Laboratory of Molecular Biology, Cambridge, United Kingdom AQ3055, strain expressing mitochondrially-targeted yellow cameleon 3.60 (YC3.60) as extrachromosomal array on pharynx, also under the *myo-2* promoter (*pmyo-2::2mt8::YC3.60*) (Alvarez-Illera et al., 2017). SJ4103 (*zcls14[myo-3::GFP(mit)]*), wild-type strain that expresses GFP at high levels in mitochondria of body wall muscle cells. Mutant strains: *daf-16(mu86), aak-2(ok524), aak-1(tm 1944);aak-2(ok524)*, *sir-2.1(ok434),* and *rsks-1(ok1255).* The strain TJ375 (*gpls1[hsp-16.2p::CFP]*), kindly supplied by Dr. Malene Hansen, Sanford Burnham Prebys Medical Discovery Institute, La Jolla, United States, was used to measure cytosolic stress. The other strains were obtained from the *Caenorhabditis* Genetics Center. Worms were maintained and handled as previously described (Stiernagle, 2006). NGM agar plates were seeded with *Escherichia coli* (OP50). Strains were maintained at 20°C.

### *C. elegans* Lifespan Assays

Lifespan assays were carried out as previously described (García-Casas et al., 2018). Briefly, eggs were obtained as described previously (Stiernagle, 2006) and transferred to *E. coli* (OP50) seeded NGM plates, either control plates or plates prepared in the presence of the required drug. For each assay, around 100 synchronized young adults (day 1) were transferred to *E. coli* (OP50) seeded NGM plates (35 mm plates, 10 worms/plate) containing 15 µM Fluorodeoxyuridine (FUdR) to avoid progeny. CGP37157 was dissolved in the NGM agar at the desired concentration. Control and drug-containing assays were always carried out in parallel. Plates were scored for dead worms every day. Worms that did not respond to touch with a platinum wire were considered dead. Age refers to days following adulthood. Plates with fungal contamination during the first 10 days of the assay were excluded from the study. Missing worms, individuals with extruded gonad or desiccated by crawling in the edge of the plate were censored. Control and drug-containing plates were kept close together in a temperature-controlled incubator set at 20°C. Statistical analysis was performed with the SPSS software, using the Kaplan-Meier estimator and the log-rank routine for significance.

### Calcium Imaging

Pharynx cytosolic and mitochondrial Ca^2+^ measurements were carried out as previously described using the strains AQ2038 and AQ3055 (Alvarez-Illera et al., 2016; Alvarez-Illera et al., 2017), at day 5 of adult life. Supplementary Figure S1 shows an image of the fluorescence of the pharynx. Body-wall muscle cytosolic [Ca^2+^] measurements were performed using the strain AQ2121 and focusing the vulva area. Briefly, worms were starved for 4–6 h before the experiments. Then, they were glued (Dermabond Topical Skin Adhesive, Johnson and Johnson) on an agar pad (2% agar in M9 buffer) and the coverslip containing the glued worm was mounted in a chamber in the stage of a Zeiss Axiovert 200 inverted microscope in the presence of 2.3 mM serotonin to stimulate pumping. Fluorescence was excited at 430 nm using a Cairn monochromator (4 nm bandwidth for the cytosolic Ca^2+^ sensor in AQ2038 and AQ2121, 7 nm bandwidth for the mitochondrial sensor in AQ3055, continuous excitation) and images of the emitted fluorescence obtained with a Zeiss C-apochromat 40 × 1.2 W objective were collected using a 450 nm long pass dichroic mirror and a Cairn Optosplit II emission image splitter to obtain separate images at 480 and 535 nm emission. The splitter contained emission filters DC/ET480/40 and DC/ET535/30 m, and the dichroic mirror FF509-FDi01–25 × 36 (all from Chroma Technology). Simultaneous 200 ms images at the two emission wavelengths were recorded continuously (2.5 Hz image rate) by a Hamamatsu ORCA-ER camera, in order to obtain 535/480 nm fluorescence ratio images values of a region of interest enclosing the pharynx terminal bulb. Experiments were performed at 20°C and carried on during 30 min of continuous recording. Fluorescence was recorded and analyzed using the Metafluor program (Universal Imaging) and a specific algorithm designed to calculate off-line the width at mid-height expressed in seconds and the height obtained as percent of ratio change of all the Ca^2+^ peaks in each experiment.

### Electropharyngeogram

Electropharyngeogram (EPG) was carried out as previously described (Álvarez-Illera et al., 2020), at day 5 of adult life. Briefly, the Nemametrix Screen Chip System (NemaMetrix, Eugene OR; Cat # SK100) with a fresh SC40 screen chip (NemaMetrix, Eugene OR; Cat # SKU: 0002) loaded with M9 buffer containing 2.3 mM serotonin, was placed on an inverted Zeiss Axiovert 200 microscope equipped with an LD A-Plan 10x objective. For each experiment, we picked 100 worms from the culture plate and washed them in 1.5 ml of 0.2 µm filtered M9 buffer + 0.1% Tween. Worms were then washed 4x with 0.2 µm filtered M9 buffer, then once in M9 buffer containing 2.3 mM serotonin, and they were finally suspended in 1 ml of M9 buffer containing 2.3 mM serotonin and allowed to settle for 15 min. All the experiments were performed between 15 and 120 min of the initial serotonin exposure. Experiments were recorded with the NemAcquire software and analyzed with the NemAnalysis v0.2 software. All the experiments with a frequency of less than 0.1 Hz or pump duration coefficient of variation bigger than 50% were rejected.

### Confocal Imaging

For confocal imaging, CGP37157 treated and untreated AQ2121 worms (for sarcomere structure evaluation) and SJ4103 (for mitochondrial organization) were transferred to a 2% agarose pad containing a drop of sodium azide 50 mM, which acts as a worm anesthetic. Then, a coverslip was used to cover the drop and worms were imaged on a Leica TCS SP5 confocal microscope. Fluorescence was excited at 488 nm, and the emission between 500 and 554 nm was collected. Images were then processed and analyzed using ImageJ software.

### Mobility Measurements

Tracking assays were performed from day 5 to day 15 of *C. elegans* AQ2121 adult worms to evaluate possible effects of CGP37157 in worm’s motility. Day 1 synchronized adult worms were placed in control 35 mm Ø plates or in plates containing 50 µM CGP37157 and kept at 20°C. Two minute videos were taken every day from day 5 to day 15 of control and treated populations, and they were analyzed offline using the ImageJ plugin “wrMTrck”, as described previously (Nussbaum-Krammer et al., 2015).

### Transcriptomic *C. elegans* Analysis

For worm synchronization, eggs were placed overnight in NGM plates without OP50 to cause L1 larvae arrest. After 12 h, L1 larvae were transferred to NGM plates seeded with OP50 and let develop at 20°C. As soon as they reach the young adult stage, worms were transferred to control and treatment (50 µM CGP37157) seeded plates, incubated at 20°C and transferred every day to new plates. At day 5 of treatment, total RNA extraction was performed. Briefly, control and treated worms were collected with distilled H_2_O, transferred to a Falcon tube and washed three times to eliminate eggs or larvae left in the plates. Then they were transferred to an eppendorf tube, the rest of the supernatant was carefully removed and three freezing-unfreezing cycles in liquid nitrogen were performed. Then, 10–20 silica beads and 500 µL of TRizol™ were added to each frozen sample, samples were homogenized using a beads homogenizer for 1 min and incubated in ice for 5 more minutes. Then 100 µL of chloroform were added to each sample, incubated for 3 min and centrifuged at 4°C, 15 min at 15.000 rpm. The upper phase was then transferred to another eppendorf tube and finally, a solution 1:1 (v/v) of the sample and Ethanol 70% was prepared. For RNA purification, the QIAGEN RNEasy Mini Kit was used. The RNA solution was transferred to a silica-membrane RNeasy spin column, washed, treated with DNases using the QIAGEN RNase-Free DNase set, and eluted using pre-heated free RNase H_2_O. Total RNA was quantified in the Nanodrop (Thermo Scientific). Global gene expression profile was analyzed at the Genomic Platform of the CIBIR (http://cibir.es/es/plataformas-tecnologicas-y-servicios/genomica-y-bioinformatica). Expression analysis was performed as described (Gómez-Orte et al., 2018; Gómez-Orte et al., 2019) using DESeq2 (Love et al., 2014) and edgeR (Robinson et al., 2010) algorithms.

## Results

We have reported previously that CGP37157 increased lifespan in *eat-2(ad1113)* mutants (caloric restriction mimetic), but it produced no effect in *daf-2(e1370)* mutants (insulin/IGF-1 receptor), *nuo-6(qm200)* mutants (mitochondrial respiratory chain complex I mutant) and *daf-15(m81)/unc-24(e138)* mutants (mTOR regulatory protein Raptor mutant) (García-Casas et al., 2019). Therefore, its effects on longevity are somehow mediated by the insulin/IGF-1 and mTOR pathways, and require functional mitochondrial respiratory chain.

**FIGURE 1:**
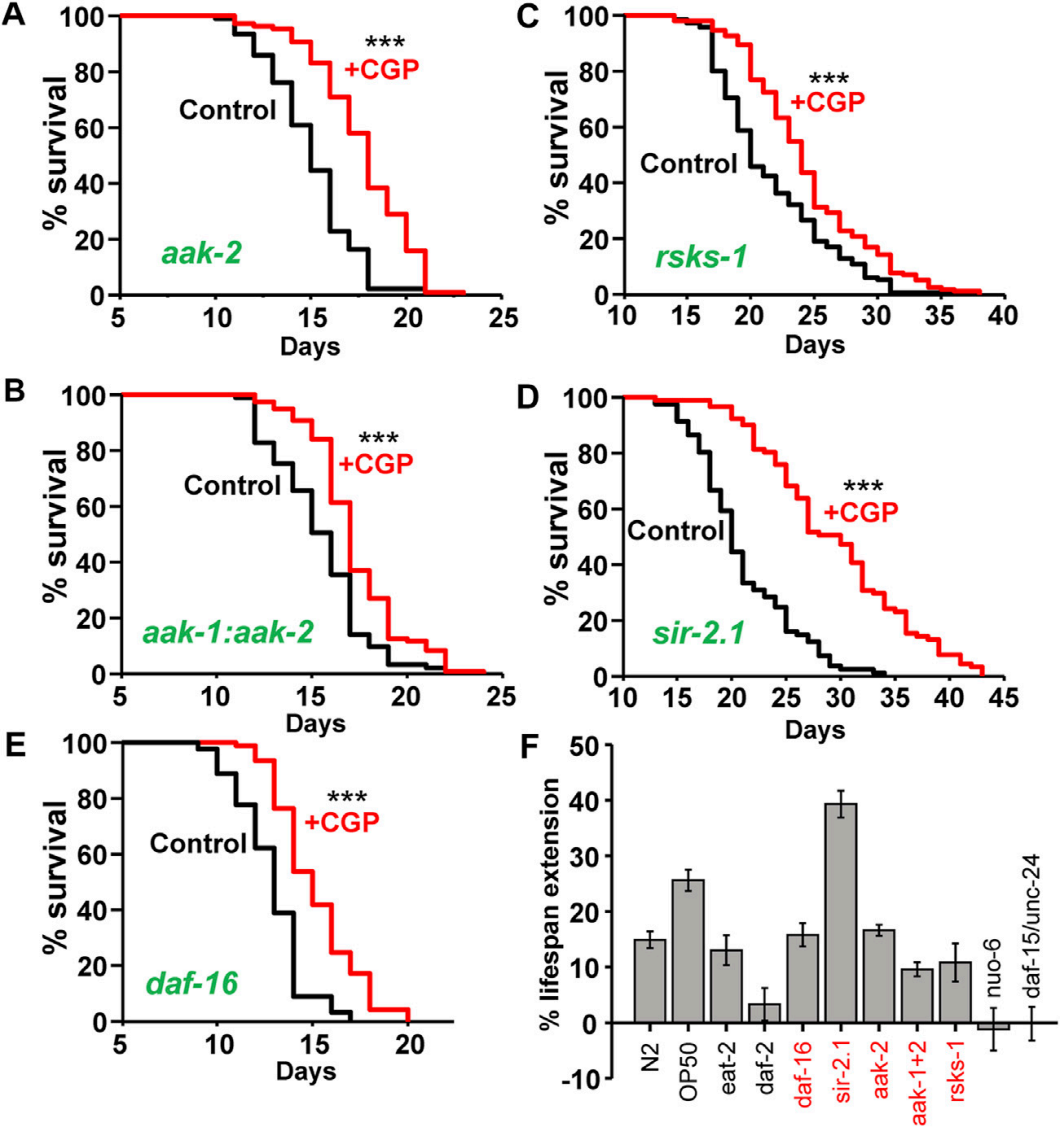
Effect of CGP37157 on the lifespan of several *C. elegans* mutants. The figure shows typical parallel control vs. drug lifespan curves obtained in different *C. elegans* mutants, as indicated in the panels. CGP37157 concentration was 50 µM. Panel **(F)** shows a summary of the mean changes in survival induced by CGP37157 in several *C. elegans* mutants, including those of Figure 1 (in red) and others reported previously (García-Casas et al., 2019). The trials shown correspond to those marked in bold in Supplementary Tables S1, S2 (more details of all the assays in Supplementary Tables S1, S2). ***p < 0.001 in the Kaplan-Meier study (log-rank routine). Supplementary Tables S1, S2 show that all the trials in every mutant found significant differences with/without CGP37157 with *p* < 0.001, except for the case of the *rsks-1* mutant, where two trials found differences significant with *p* < 0.001, one with *p* < 0.05 and one found no significant differences.

To extend our genetic analysis and define which pathways are relevant for the lifespan extension mediated by CGP37157, we scored the effect of this compound on the lifespan of mutants of several additional pathways related to longevity. Figure 1 shows that CGP37157 increased lifespan in a similar way in mutants of the AMP-activated kinase, both in mutants of the catalytic subunit α2 (*aak-2(ok524)*, panel a) and in double mutants of both catalytic *α* subunits (*aak-1(tm 1944);aak-2(ok524)*, panel b). CGP37157 was also effective increasing the lifespan in mutants of the mTOR substrate S6 kinase (*rsks-1(ok1255)*, panel c), in mutants of the sirtuin pathway (*sir-2.1(ok434)*, panel d), and in mutants of the transcription factor *daf-16(mu86)*/FOXO (panel e). These pathways are therefore not involved in the lifespan increase induced by CGP37157. Figure 1F, shows a summary of the effects of this compound in the different mutants (see also Supplementary Tables S1, S2).

**FIGURE 2:**
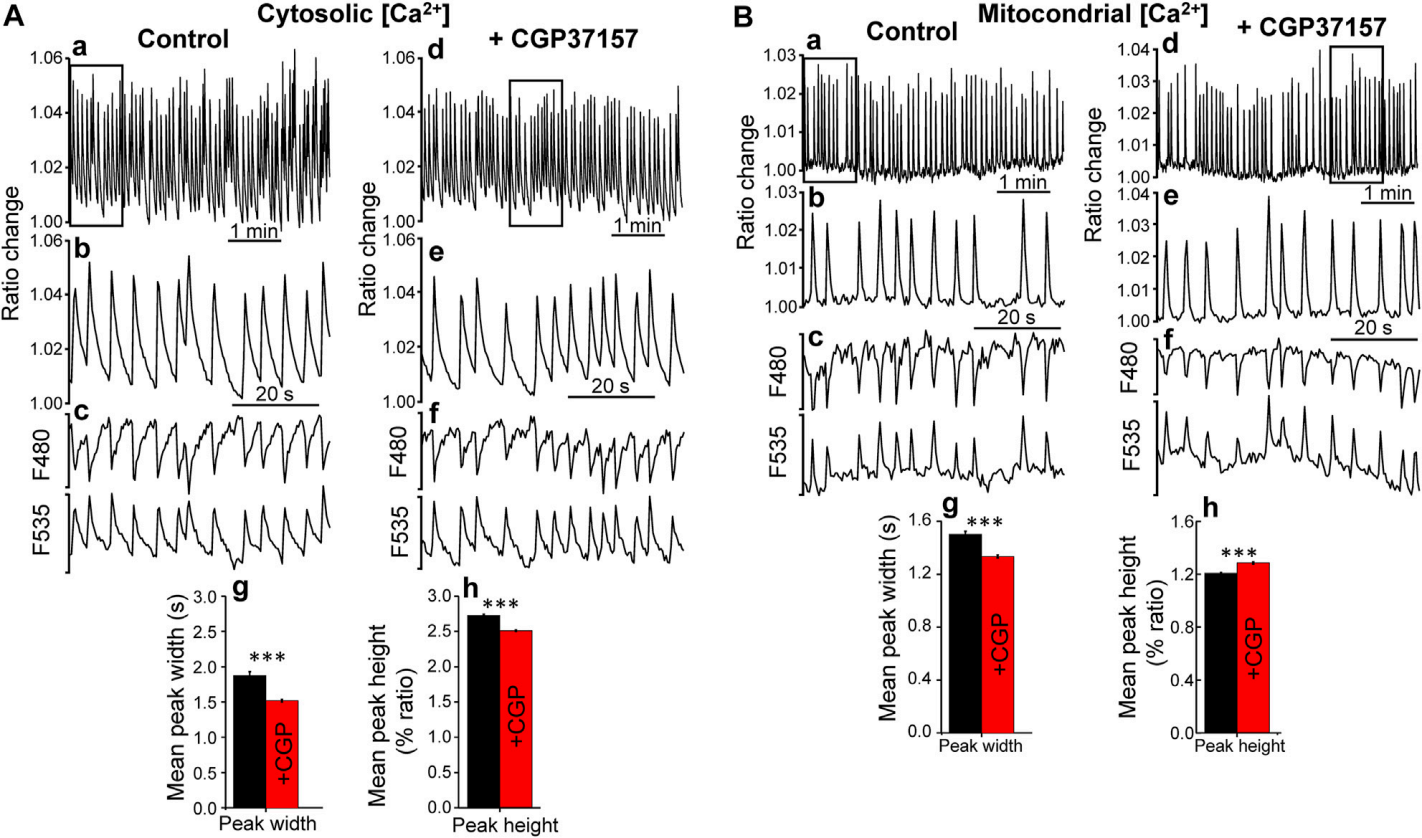
Effect of CGP37157 on *C. elegans* pharynx cytosolic **(A)** and mitochondrial **(B)** [Ca^2+^]. Panels a and d show 5 min of typical records of cytosolic **(A)** or mitochondrial **(B)** [Ca^2+^] oscillations obtained from *C. elegans* worms at day 5 of adult life either treated (panel d) or untreated (panel a) with 50 µM CGP37157 since day 1 of adult life. Panels b and e show expanded the 1 min region marked with a square in the 5 min record of the panel above. Panels c and f show the single wavelength records with emission at 480 and 535 nm corresponding to the 1 min ratio records of the panels above, showing the expected mirror changes. Panels g and h show the mean peak width and mean peak height of pharynx cytosolic **(A)** or mitochondrial **(B)** [Ca^2+^] oscillations. ***p < 0.001, ANOVA test, *n* = 5,009 to 8,456 peaks analyzed.

In order to investigate the mechanism involved in the increase in lifespan induced by this compound, we performed measurements of cytosolic and mitochondrial Ca^2+^ dynamics in the pharynx of live worms, either treated or untreated with CGP37157. As we have reported previously, the pharynx displays a persistent fast Ca^2+^ oscillatory activity that can be followed for long time periods, both in the cytosol (Alvarez-Illera et al., 2016) and in the mitochondria (Alvarez-Illera et al., 2017). We have made an analysis of all the cytosolic and mitochondrial Ca^2+^ peaks recorded in a series of N2 wild-type worms in every condition. Figure 2A shows typical records of the cytosolic [Ca^2+^] peaks obtained in the absence and in the presence of CGP37157. The dynamics of [Ca^2+^] oscillations was quite similar, although we could detect a 20% decrease in the mean peak width and 8% decrease in the mean peak height in worms treated with CGP37157. Instead, when we studied the mitochondrial Ca^2+^ peaks, the mean peak width was reduced by 11% but the mean peak height was increased by 7% (Figure 2B). In addition, to assess the effect of CGP37157 on pharynx muscle contraction, we made electrical measurements of pharynx contraction (Electropharyngeogram, EPG). Supplementary Figure S2 shows that the presence of CGP37157 did not induce statistically significant changes in the main EPG parameters, although the mean values shifted in the same direction that the changes in [Ca^2+^], namely, a decrease in pump duration.

**FIGURE 3:**
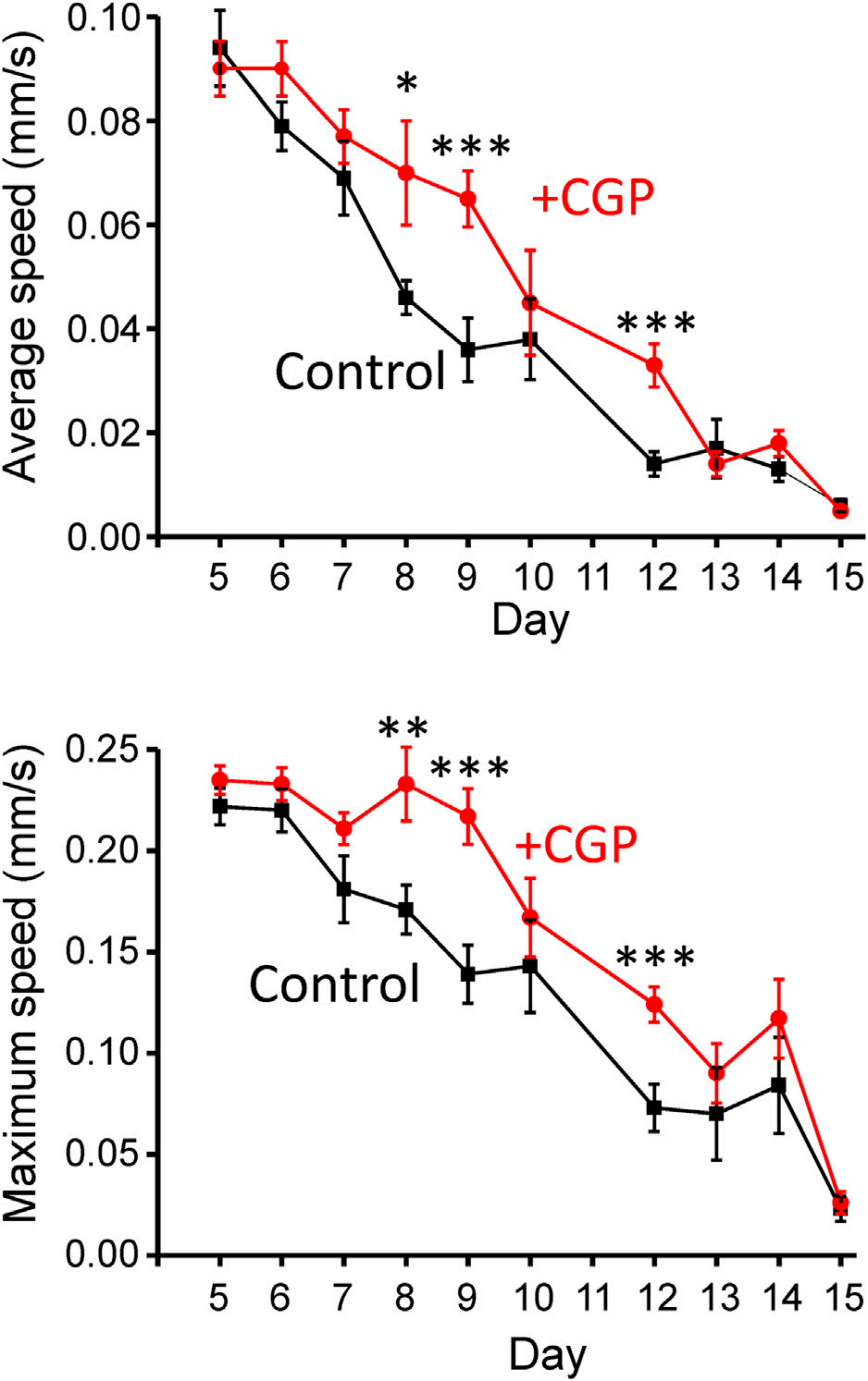
Effect of CGP37157 on the mobility of the worms. Mobility was estimated from 2 min videos analyzed with the “wrMTrck” ImageJ plugin to obtain the mean average and maximum speed, as described in Methods. *p < 0.05; **p < 0.01; ***p < 0.005, ANOVA test, *n* = 9–15 worms per condition.

An important point regarding the effects of drugs that modify the lifespan concerns their effects on functional parameters indicative of good health. We have studied here the effect of CGP37157 on the mobility of the worms in tracking experiments. Figure 3 shows the mean average and maximum speed obtained from a series of tracking experiments performed at different days of adult life. The data show that CGP37157 increased the mean average and maximum speed of the worms, an effect that was significant in the interval between days 8–12.

**FIGURE 4:**
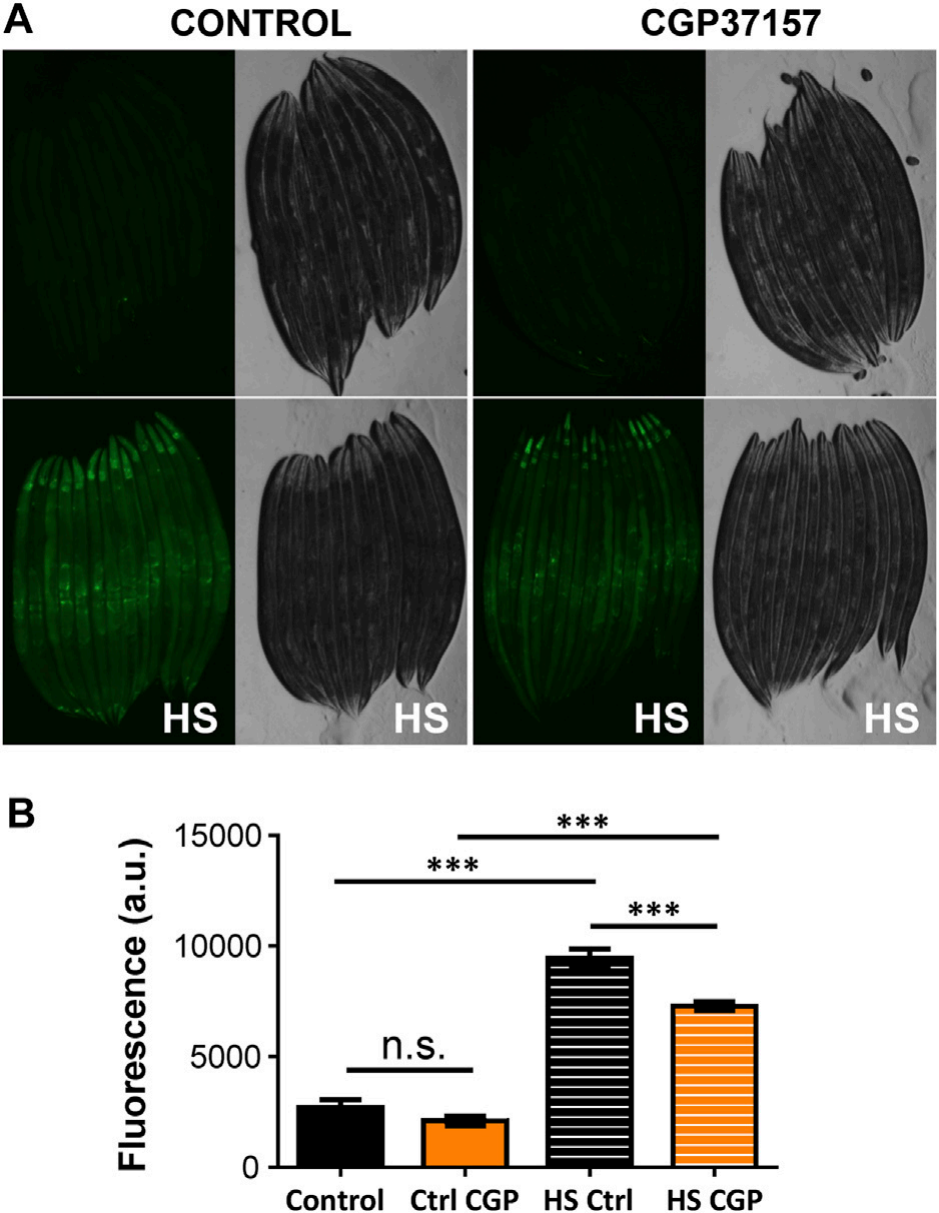
Effect of CGP37157 on cytosolic stress. *hsp-16.2* expression was evaluated at day 3 of adult life using the strain TJ375, that induces GFP expression when *hsp-16.2* is transcribed. **(A)** shows the mean fluorescence obtained before **(upper panels)** and after **(lower panels)** a temporary heat shock (HS) treatment of 2 h at 35°C followed by overnight recovery, either in the absence **(left panels)** or in the presence **(right panels)** of 50 µM CGP37157. **(B)** shows the mean fluorescence obtained in each condition compared with the ANOVA test (*n* = 23 to 37 worms/condition. ***p < 0.001).

We have also studied the effect of CGP37157 on cellular stress, which is considered to be a very important phenomenon involved in the toxicity of many drugs, in addition to other mechanisms such as mitochondrial dysfunction or oxidative stress. For that, we have monitored the expression of *hsp-16.2*, a heat shock protein related to stress resistance that is induced by a number of types of cellular stress, including heat shock or oxidative stress (Link et al., 1999). Figure 4 shows that expression of this marker largely increased after a heat sock, but was not modified by treatment with CGP37157. This suggests that the effects of CGP37157 on lifespan are not mediated by an increase in the expression of stress resistance proteins.

**FIGURE 5:**
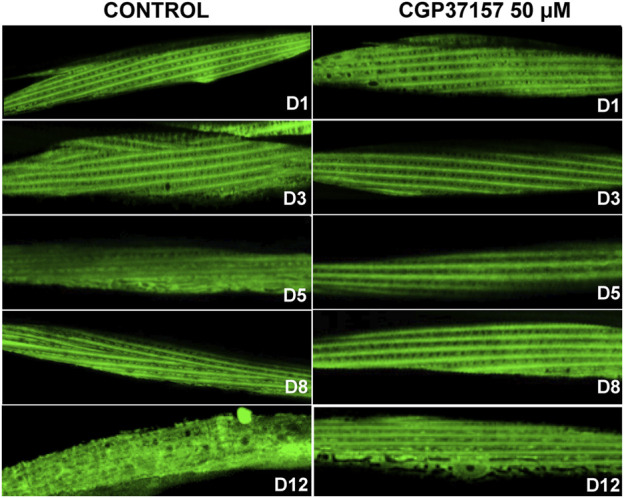
Effect of CGP37157 on the sarcomeric structure in *C. elegans* body wall muscle during aging. AQ2121 worms, expressing the YC2.1 cameleon probe in the cytosol, were imaged by confocal microscopy at different days of adult life, either treated or not with 50 µM CGP37157 since day 1 of adult life.

Another functional parameter we have monitored is the sarcomere structure in body wall muscle cells. It has been reported that sarcomere structure of body wall muscle cells undergoes a progressive decline with age, so that sarcomere lose their densely packed structures and regular and parallel orientations as aging progresses, a phenomenon typical of sarcopenia (Herndon et al., 2002; Tiku et al., 2016). Figure 5 shows the effect of CGP37157 on sarcomere structure at several days of adult life. There are no differences at day 1 of adulthood, indicating that worms began the treatment in similar conditions. On day 3 we see that control worms begin to lose part of the parallel structure while the treated worms continue to show regular orientations. This effect is even more evident in the representative images of day 5, day 8, and day 12 of treatment of Figure 5. More images at day 8 are shown in Supplementary Figure S3. Control worms start to lose either density or the sarcomeric regular distribution, while treated worms still maintain a similar structure to that observed at day 1 of adulthood. Thus, treatment with CGP37157 delays muscle decline in *C. elegans* and favors the maintenance of the sarcomeric structure in body wall muscle cells.

**FIGURE 6:**
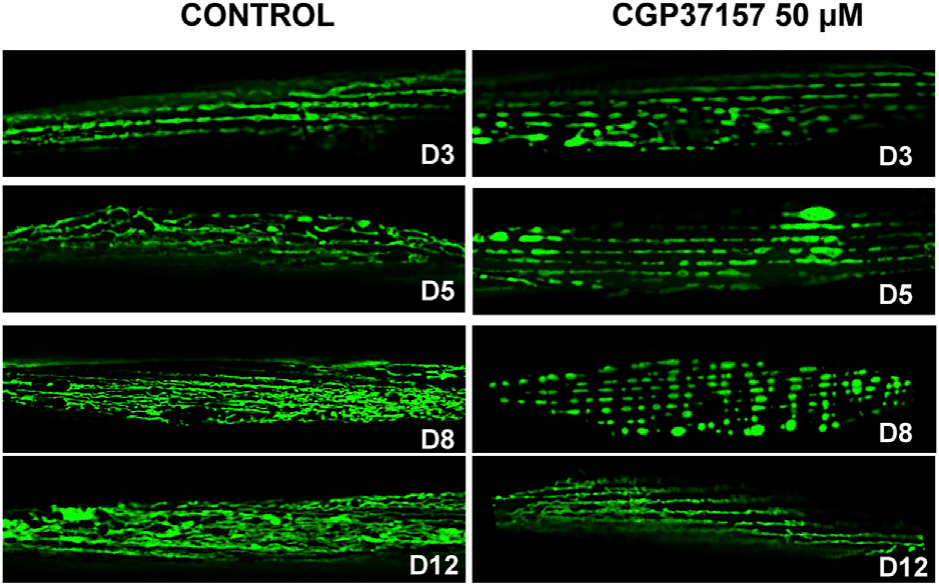
Effect of CGP37157 on the mitochondrial structure in *C. elegans* body wall muscle during aging. SJ4103 worms, expressing GFP inside mitochondria of body wall muscle cells, were imaged by confocal microscopy at different days of adult life, either treated or not with 50 µM CGP37157 since day 1 of adult life.

It has been recently described that alterations in muscle mitochondrial structure are strongly correlated with the decline of both sarcomeric structure and speed of movement (Gaffney et al., 2018). We have therefore studied the effects of the treatment with CGP37157 on mitochondrial structure in *C. elegans* body wall muscle cells. For that, we have used the strain SJ4103, which expresses the fluorescent protein GFP targeted to mitochondria in body wall muscle cells under the *myo-3* promoter. Figure 6 shows the evolution of mitochondrial structure in body wall muscle cells during aging, both in the absence and in the presence of CGP37157. The control images show clearly that mitochondrial structure undergoes a decline as the organism age. At day 3, the mitochondrial structure is quite similar to that observed in the sarcomeric structure, indicating the high association present between ER and mitochondria in these cells. This association weakens during aging, causing this well-organized structure to be lost. When comparing the images of the control group with those of the CGP37157-treated group, it is clear that the treatment favors the maintenance of the mitochondrial structure with age. Even at day 12, although the structure is not as well preserved as at day 8, a parallel mitochondrial distribution is still apparent in the treated worms. More images at day 8 are shown in Supplementary Figure S4.

**FIGURE 7:**
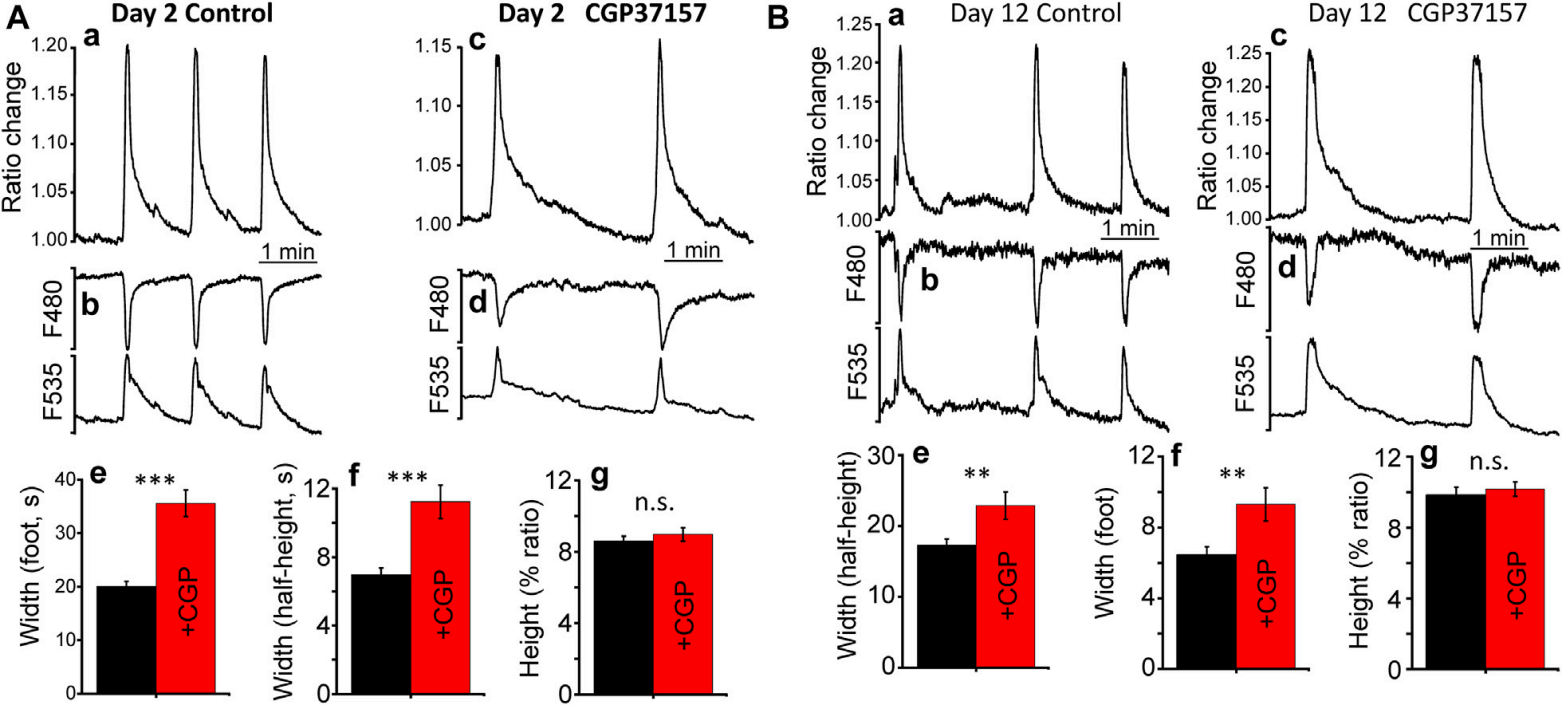
Effect of CGP37157 on *C. elegans* vulva cytosolic [Ca^2+^] at day 2 **(A)** and day 12 **(B)** of adult life. Panels a and c show 5 min of typical records of spontaneous cytosolic [Ca^2+^] oscillations obtained from *C. elegans* worms at day 2 of adult life either treated (panel c) or untreated (panel a) with 50 µM CGP37157 since day 1 of adult life. Panels b and d show the single wavelength records with emission at 480 and 535 nm corresponding to the 5 min ratio records of the panels above, showing the expected mirror changes. Panels e. g., and h show the mean peak width at the foot of the peak, the mean peak width at half-height and the mean peak height of the vulva cytosolic [Ca^2+^] oscillations. ***p < 0.001; **p < 0.01; ANOVA test, *n* = 113 to 190 peaks analyzed.

Because of the decline we see in worm mobility and body-wall muscle sarcomere and mitochondrial structure, we have also investigated the effect of CGP37157 on body-wall muscle cytosolic [Ca^2+^] dynamics at both day 2 and day 12. We used the AQ2121 strain (expressing the [Ca^2+^] sensor YC2.1 in body-wall muscle and vulva muscle) and monitored the vulva region, which shows spontaneous large [Ca^2+^] oscillations even in the glued worms. Although the function of these contractions is to expel the eggs, they are present throughout the worm's life. Figure 7A shows the [Ca^2+^] dynamics observed at day 2, both in the absence and in the presence of CGP37157. [Ca^2+^] peaks were much larger and wider than those measured in the pharynx, and the presence of CGP37157 did not modify the height of the peaks, but produced a large increase in the mean width, both in the width measured at the foot of the peaks and in the width measured at half-height. Similar results were obtained at day 12 (Figure 7B). Although the magnitude of the effects was smaller at day 12, the presence of CGP37157 significantly increased also the width of the peaks, and did not modify the height.

**FIGURE 8:**
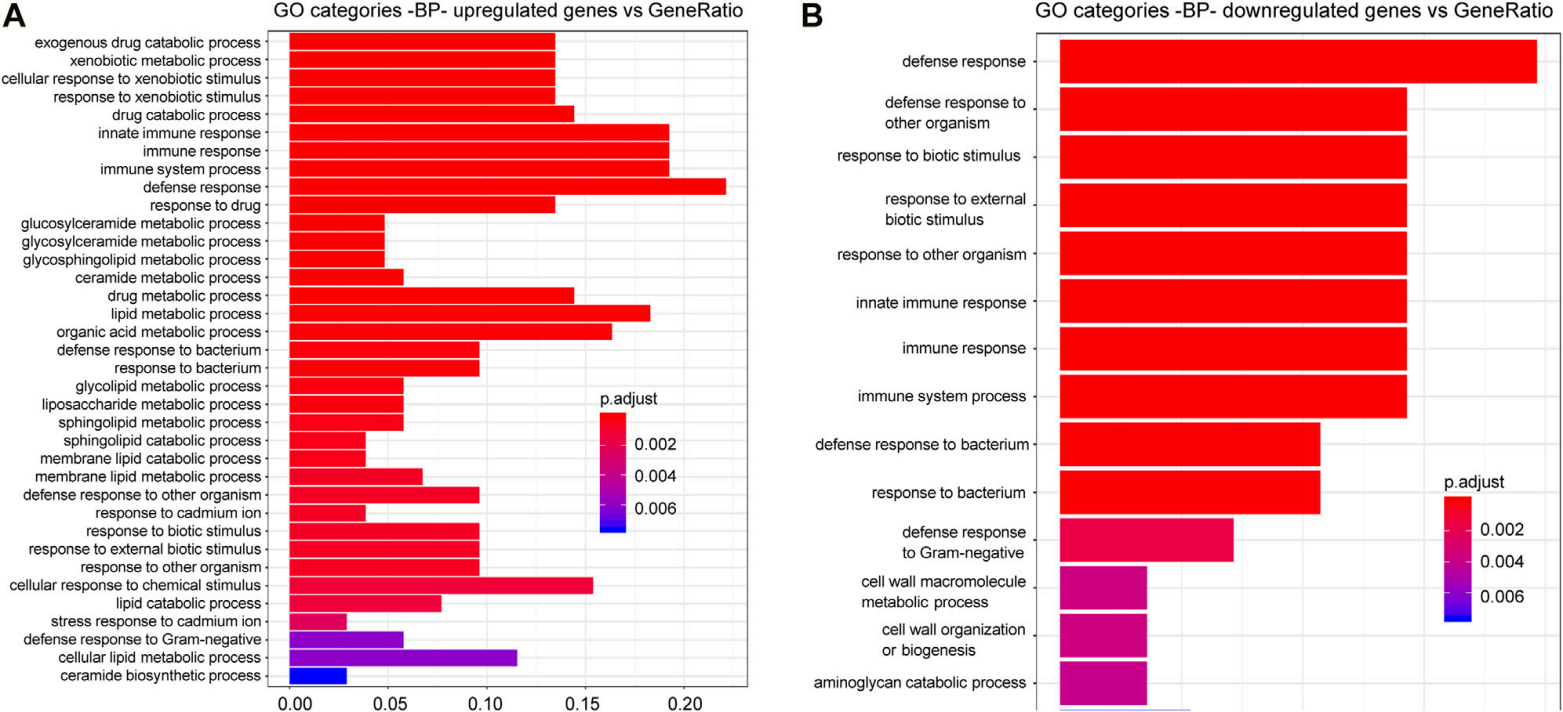
*C. elegans* gene up-regulation and down regulation in the presence of CGP37157. Gene Ontology (GO) Biological Processes (BP) vs. number of genes within each category appears represented in color bars, 1 bar per GO term. The length of the bars indicates the number of genes belonging to the different GO categories and the color indicates the statistical significance, from those with highly significant expression difference (red) to those with lower significance (blue). Gene expression data were normalized by a negative binomial distribution model by using DESeq2 and EdgeR implementations and compared in pairs to analyze the effect of CGP37157 treatment. Common up-regulated **(A)** and down-regulated **(B)** genes determined by both methodologies were used for annotation and GO analyses.

Finally, to obtain further information on the functional effects induced by CGP37157 treatment, we have studied the changes induced by this treatment on the global gene expression pattern. Analysis was performed as described previously (Gómez-Orte et al., 2018). Most of the GO categories showing significant changes correspond to cellular response to exogenous drugs, immune and defense response and several lipid metabolic processes (Figure 8; Supplementary Tables S3, S4). Many of these changes likely reflect adaptations by *C. elegans* to the presence of the xenobiotic CGP37157. However, it is interesting to note the presence of several consistent changes in lipid metabolism enzymes, which could be relevant to the effect of this compound on lifespan. We find here that CGP37157 induced a 2-4-fold increase in expression of the lysosomal lipases *lipl-1*, *lipl-2,* and *lipl-5*, and the fasting-induced lipase *fil-1*. Conversely, the expression of the acyl-CoA dehydrogenases *acdh-1*, *acdh-2,* and *acdh-9*, the acyl-CoA oxidase *acox-3*, the long chain acyl-CoA synthetase *acs-3*, and the desaturases *fat-5* and *fat-7* were reduced to less than half. There was also a global increase in the expression of ceramide metabolism enzymes, including glucosyl-ceramidases *gba-1*, *gba-2,* and *gba-4*, ceramide glucosyl-transferases *cgt-1,* and *cgt-2*, and the sphingomyelin phosphodiesterase *asm-3*.

In addition, looking carefully at the list of genes showing significant changes in the presence of this compound, we could detect *ncx-6*, one of the mitochondrial Na^+^/Ca^2+^ exchanger genes, which increased expression 4-fold with very high significance. Of the rest of Na^+^/Ca^2+^ exchanger genes, only *ncx-9* showed a significant change, but with smaller significance and only when analyzed using DESeq2 algorithm, not with EdgeR algorithm. No other gene related to calcium homeostasis showed significant differences.

## Discussion

In this work, we have further explored the mechanism of the increased lifespan induced by compound CGP37157 in *C. elegans* worms. First, we have studied the effect of this compound in several mutants of pathways related to longevity. Together with studies performed in other mutants reported previously (García-Casas et al., 2019), our results provide a good picture of the pathways involved in the effect. The increase in lifespan induced by CGP37157 in the following mutants: *eat-2(ad1113), daf-16(mu86), sir-2.1(ok434), aak-2(ok524), aak-1(tm 1944);aak-2(ok524),* and *rsks-1(ok1255)* remained similar to that obtained in N2 wild-type controls. Instead, CGP37157 was ineffective in *daf-2(e1370), nuo-6(qm200),* and *daf-15(m81)/unc-24(e138)* mutants. In the first case, when the effect remains unchanged, this means that the pathways inactivated in these mutants are not relevant for the effect of the compound. On the contrary, when the lifespan increase disappears, this is evidence that those pathways are required for the effect.

The *eat-2* mutant has increased longevity due to caloric restriction, and thus the effect of CGP37157 is not mediated by caloric restriction. *daf-16* is a transcription factor that activates several genes that promote longevity, and *daf-2* is the insulin/IGF-1 receptor, whose activation leads to inactivation of *daf-16* by cytoplasmic sequestration. Our results suggest that CGP37157 may activate directly some longevity mechanism downstream of *daf-16*. In that case, it could be activated in *daf-16* mutants (in the absence of DAF-16), but it would disappear in *daf-2* mutants because *daf-16* is maximally activated in those mutants. One of the downstream targets of *daf-16* is the TORC1 coactivator *daf-15/Raptor*, and DAF-16/FOXO inhibits the expression of *daf-15/Raptor* (Sun et al., 2017). Given that CGP37157 was also ineffective in *daf-15/raptor* mutants, the DAF-16-sensitive TORC1 pathway could be essential for the effect of CGP37157. Instead, sirtuins and AMP-activated kinase are not involved. Also, the TORC1 substrate S6 kinase is not required, as CGP37157 is still effective in *rsks-1* mutants. Finally, the presence of a functional mitochondrial respiratory chain is necessary, because the compound was ineffective in the respiratory chain complex I mutant *nuo-6*. In summary, the increase in lifespan induced by CGP37157 is probably mediated by TORC1 inhibition and requires functional mitochondrial respiration to develop.

We have then monitored cytosolic and mitochondrial [Ca^2+^] oscillations in the worm pharynx both in the presence and in the absence of CGP37157. Although oscillatory activity was similar, there were small but significant changes in the shape and frequency of the [Ca^2+^] peaks. Treatment with CGP37157 reduced both the width and the height of the cytosolic [Ca^2+^] peaks. Instead, it reduced the width but increased the height of the mitochondrial [Ca^2+^] peaks (Figure 2). The electropharyngeogram showed a decrease in pump duration that was consistent with the changes in [Ca^2+^], although it was not statistically significant, probably because of the large variability of the pump electrical recordings. The decrease in the width and height of the cytosolic [Ca^2+^] peaks may be explained by inhibition of Ca^2+^ entry through either a voltage-dependent Ca^2+^ channel (such as EGL-19) or the CLHM-1 channel. CGP37157 has been shown to inhibit the mammalian homologues of these channels (Baron and Thayer, 1997; Moreno-Ortega et al., 2015). In the case of mitochondrial [Ca^2+^], the increase in the height of the peaks may be a direct consequence of the inhibition of the mitochondrial Na^+^/Ca^2+^ exchanger (the main mitochondrial Ca^2+^-exit pathway) by CGP37157, which would explain the differential behavior of Ca^2+^ dynamics in both compartments. However, inhibition of mitochondrial Ca^2+^ exit should have increased also the width of the mitochondrial [Ca^2+^] peaks. A possible explanation may rely in the small magnitude of the cytosolic and mitochondrial [Ca^2+^] peaks in the pharynx. We have recently calculated that the pharynx spontaneous cytosolic [Ca^2+^] peaks are no higher than 200 nM (García-Casas et al., 2021) and ratio changes suggest that mitochondrial [Ca^2+^] peaks should be no higher than that. Given that the mNCX has low Ca^2+^ affinity (Paucek and Jabůrek, 2004), other CGP37157-insensitive Ca^2+^ extrusion mechanisms may be responsible for the return to resting levels of the spontaneous oscillations we measure in the pharynx. Instead, in the case of vulva muscle, the spontaneous [Ca^2+^] peaks are much larger and we can see an increase in the width of the cytosolic [Ca^2+^] peak in the presence of CGP37157. This effect can be attributed to the inhibition of mNCX, which would increase the width of the mitochondrial [Ca^2+^] peak and the slow Ca^2+^ release from mitochondria would then increase also the width of the cytosolic [Ca^2+^] peak. In summary, CGP37157 may be acting via two mechanisms: inhibition of the mNCX to reduce mitochondrial Ca^2+^ release, whenever the [Ca^2+^] peak is large enough to activate mNCX, and inhibition of a plasma membrane Ca^2+^ channel to reduce Ca^2+^ entry. The effects on longevity must come from a combination of both effects on the different cell types.

We have also investigated the effects of CGP37157 on several functional parameters representative of good health. This is an important aspect, because not all the increases in lifespan correlate with an improvement in healthspan. In this sense, several possible biomarkers of aging have been proposed in *C. elegans* including physiological markers such as locomotion (Huang et al., 2004; Hsu et al., 2009; Pincus et al., 2011; Hahm et al., 2015), pharyngeal pumping rate (Huang et al., 2004), progeny number (Pickett et al., 2013), cellular markers such as muscle sarcomeric structure (Herndon et al., 2002; Tiku et al., 2016) or mitochondrial structure (Gaffney et al., 2018), and molecular markers like stress proteins, for example, expression of *hsp-16.2* (Rea et al., 2005). In this work, several of these biomarkers of aging have been studied in *C. elegans*, and all of them indicate that worms treated with CGP37157 are in better health conditions than the untreated ones.

One of the physiological biomarkers studied in this work was locomotion. It has been widely described that an improvement in locomotion, either in worms that display fast locomotion during early adulthood (Huang et al., 2004; Pincus et al., 2011; Hahm et al., 2015), or in worms that maintain their youth speed during middle age (Hsu et al., 2009), correlate positively with lifespan. When the effects of CGP37157 on *C. elegans* locomotion were studied, both their average speed (mm/s) and their maximum speed (mm/s) were increased in middle age worms, demonstrating that CGP37157 does not only extends *C. elegans* lifespan, but it also improves their locomotion capacity, and therefore their muscular function.

Among the molecular markers that have been described, the expression of *hsp-16.2* has been evaluated after the treatment with CGP37157. This chaperone is a marker of cytosolic stress and it has been proven to correlate in a positive manner with aging (Rea et al., 2005). To assess the possible changes in *hsp-16.2* expression with the treatment, the strain TJ375 was used, as GFP expression in this strain is driven by the *hsp-16.2* promoter. The results show that the treatment with CGP37157, at day 3 of adulthood, did not produce changes in GFP expression in this worms, thus no cytosolic stress was induced by the treatment.

We have also investigated the effects of CGP37157 on the development of sarcopenia in body wall muscle. Sarcopenia is defined as a progressive loss of muscle mass with advancing age characterized by a decline in muscle quantity and quality (Evans, 1995; Roubenoff and Hughes, 2000). The molecular mechanisms behind the development of sarcopenia remain poorly defined. However, gene expression studies investigating human muscle with age have suggested that alterations in various metabolic pathways including the electron transport chain, the insulin signaling pathway and the mTOR pathway take part in the development of this process (Zahn et al., 2006; Phillips et al., 2013; Tang et al., 2019). Specifically, the inhibition of mTORC1 in aging mouse induces gene expression changes that reduce oxidative stress and muscle fiber damage and loss (Tang et al., 2019). Moreover, it has been recently postulated that mitochondrial deterioration in muscle and motor neurons is the primary initiator of sarcopenia and that interventions aimed at improving mitochondrial function and proteostatic maintenance could mitigate or treat this process of muscle loss (Alway et al., 2017; Coen et al., 2019).

The sarcopenia process has been demonstrated to be conserved in *C. elegans*, and recently a greater loss of mitochondrial function with aging has been associated with an earlier onset of sarcopenia in *C. elegans* (Gaffney et al., 2018). Also, the insulin signaling pathway appears to be involved in this phenomenon, because *daf-2* mutants are resistant to the development of sarcopenia and associated declines in motility during aging (Duhon and Johnson, 1995; Herndon et al., 2002; Glenn et al., 2004). Due to the lack of effect of CGP37157 treatment in *daf-2* and *daf-15* mutants, and the improved locomotion of the treated worms, we decided to investigate the possible effects of CGP37157 in sarcopenia and mitochondrial organization in *C. elegans* body wall muscle cells.

Our results show that treatment with CGP37157 is able to delay the sarcopenia process in *C. elegans* worms, maintaining the parallel sarcomeric structure of body wall muscle cells of treated worms when compared to the control group. Moreover, mitochondrial distribution and morphology was also improved in the worms under the CGP37157 treatment. Since loss-of-function mutations in *daf-2* and *daf-15* blocked the effect of CGP37157, these beneficial effects in muscle integrity and maintenance may be due to the negative modulation by CGP37157 of both the insulin and mTOR pathways. Interestingly, in spite of the progressive decline in muscle structure and function with age, we found little changes in [Ca^2+^] dynamics in vulva muscle at day 2 and day 12. We have reported before a similar effect when studying [Ca^2+^] dynamics in the pharynx during aging. While pharynx pumping is drastically reduced with age, [Ca^2+^] dynamics was much better preserved (Alvarez-Illera et al., 2016)**.**

Finally, the transcriptomic study detected that CGP37157 induced significant changes in a series of genes related to the cellular response to exogenous drugs, immune and defense response and lipid metabolic processes. While changes in the expression of genes related to response to xenobiotics were expected, the changes observed in lipid metabolism enzymes may perhaps be more relevant for its effect in lifespan. Many changes in *C. elegans* lipid metabolism have been previously associated to longevity (O’Rourke and Ruvkun, 2013; Kim et al., 2016; Lemieux and Ashrafi, 2016; Imanikia et al., 2019; Johnson and Stolzing, 2019). This includes changes in the levels of specific lipases (Buis et al., 2019; Murphy et al., 2019), desaturases (Maulik et al., 2019), the acyl-CoA synthetase *acs-3* (Ward et al., 2014) and ceramide metabolism (Kim et al., 2016; Cui et al., 2017; Johnson and Stolzing, 2019), but a full pattern of survival-promoting changes in lipid metabolism has not yet been defined. Some of the changes we observed are consistent with those previously described to promote survival, but it is difficult to know whether they are actually related or not to the increase in longevity induced by CGP37157.

**FIGURE 9:**
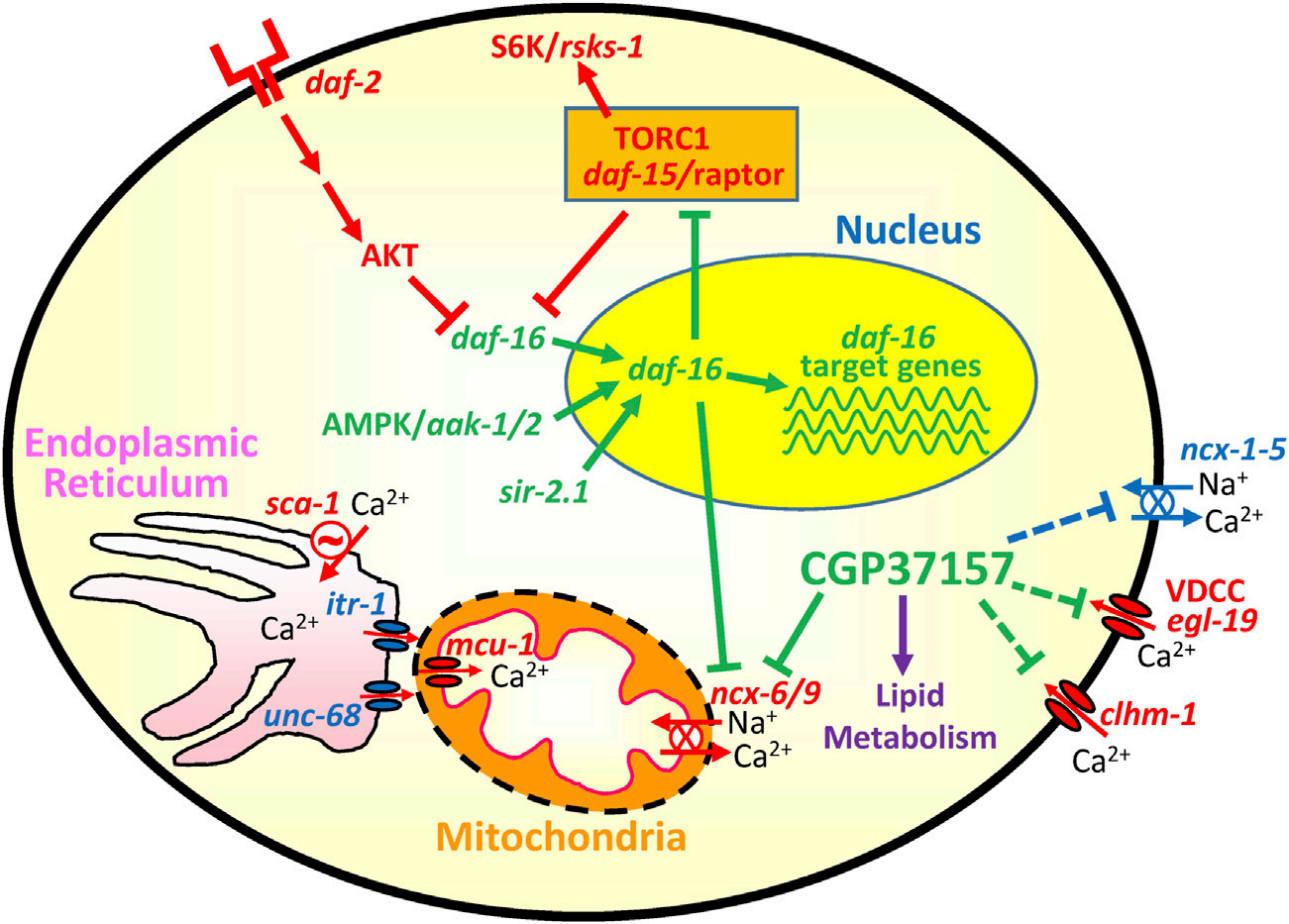
Cartoon showing the main pathways involved in the effect of CGP37157. Activation of the *daf-2* receptor leads to inactivation of *daf-16* by cytoplasmic sequestration. When this pathway is downregulated, daf-16 enters the nucleus and regulates a series of target genes that increase longevity. AMP-activated kinase and SIR-2.1 promote *daf-16* activation, while the TORC1 complex inhibits it. Among the downstream targets of *daf-16* we have the TORC1 coactivator *daf-15*/Raptor and also the mitochondrial Na^+^/Ca^2+^ exchanger *ncx-6.* Both are downregulated by *daf-16*. CGP37157 also inhibits *ncx-6/ncx-9*, and it may also inhibit several Ca^2+^ channels and transporters in the plasma membrane. The cartoon also includes the Ca^2+^ connection between endoplasmic reticulum and mitochondria, composed of the Ca^2+^ channels *itr-1* (IP_3_ receptor) and *unc-68* (ryanodine receptor), the ER Ca^2+^ pump (*sca-1*) and the mitochondrial Ca^2+^ uniporter (*mcu-1*). In green, pathways pro-survival. In red, pathways pro-aging.

In addition, we found in the transcriptomic study one gene involved in Ca^2+^ homeostasis that underwent a highly significant change. That was *ncx-6*, one of the 10 genes corresponding to Na^+^/Ca^2+^ exchangers present in the *C. elegans* genome, whose expression increased 4-fold in the presence of CGP37157. *ncx-6* belongs to the NCLX family, which in humans has only one representative and in *C. elegans* includes *ncx-6, ncx-7, ncx-8, ncx-9* and *ncx-10* (Sharma et al., 2013; He and O’Halloran, 2014; Sharma and O’Halloran, 2014)*.* Human NCLX is a Na^+^/Ca^2+^ exchanger located in the inner mitochondrial membrane, and it is the main target of CGP37157. We would therefore expect that the NCLX representatives in *C. elegans* would also catalyze CGP37157-sensitive Na^+^/Ca^2+^ exchange in the inner mitochondrial membrane. In fact, that has already been shown for *ncx-9* (Sharma et al., 2017), but not yet for *ncx-6*. On the other hand, it has been reported that *ncx-6* is the only DAF-16 target gene within the *ncx* family, and it is downregulated by DAF-16 (Tepper et al., 2013). This is consistent with our data because both DAF-16 activation and CGP37157 increase lifespan and reduce NCX-6 activity, by downregulation or by inhibition, respectively. The role of NCX-6 in this phenomenon requires further study. Finally, the increase in expression of *ncx-6* induced by CGP37157 treatment could be explained as a compensatory response to the inhibition by CGP37157, but further work should be necessary to prove that. In any case, we must keep in mind that the mitochondrial localization of *ncx-6* has not yet been demonstrated, and that the only *C. elegans* Na^+^/Ca^2+^ exchanger with CGP37157-sensitive mitochondrial transport activity known to date is *ncx-9*. Figure 9 shows a cartoon explaining the main pathways that could be involved in the mechanism of action of CGP37157.

Our results indicate that CGP37157 modulates Ca^2+^ homeostasis by acting on mitochondrial Na^+^/Ca^2+^ exchangers (with the NCX-6 isoform playing an important role) and plasma membrane Ca^2+^ channels, and it is able to increase longevity by a mechanism involving mitochondria and mTOR. This results add on our previous reports of the increase in lifespan induced by the inhibition of the Sarco Endoplasmic Reticulum Ca^2+^ Pump (García-Casas et al., 2018; García-Cases et al., 2021). Both Ca^2+^ pathways are amenable to pharmacological intervention and perhaps other Ca^2+^ transport systems in *C. elegans*, e.g., the L-type voltage-dependent Ca^2+^ channel *egl-19* (Kwok et al., 2006), may also be interesting targets to act on them pharmacologically to increase longevity. Thus, Ca^2+^ signaling pathways may offer new targets for acting on aging.

## Data Availability

The datasets presented in this study can be found in online repositories. The names of the repository/repositories and accession number(s) can be found below: www.ncbi.nlm.nih.gov/geo/, GSE173646
