# Supplementary material for: The Mitochondrial Na+/Ca2+ Exchanger Inhibitor CGP37157 Preserves Muscle Structure and Function to Increase Lifespan and Healthspan in Caenorhabditis elegans
Source: Front Pharmacol. 2021 Jun 15;12:695687. doi: 10.3389/fphar.2021.695687 (PMC8241105; doi:10.3389/fphar.2021.695687)
Supplement: Supplementary file 3 [file DataSheet1.PDF]

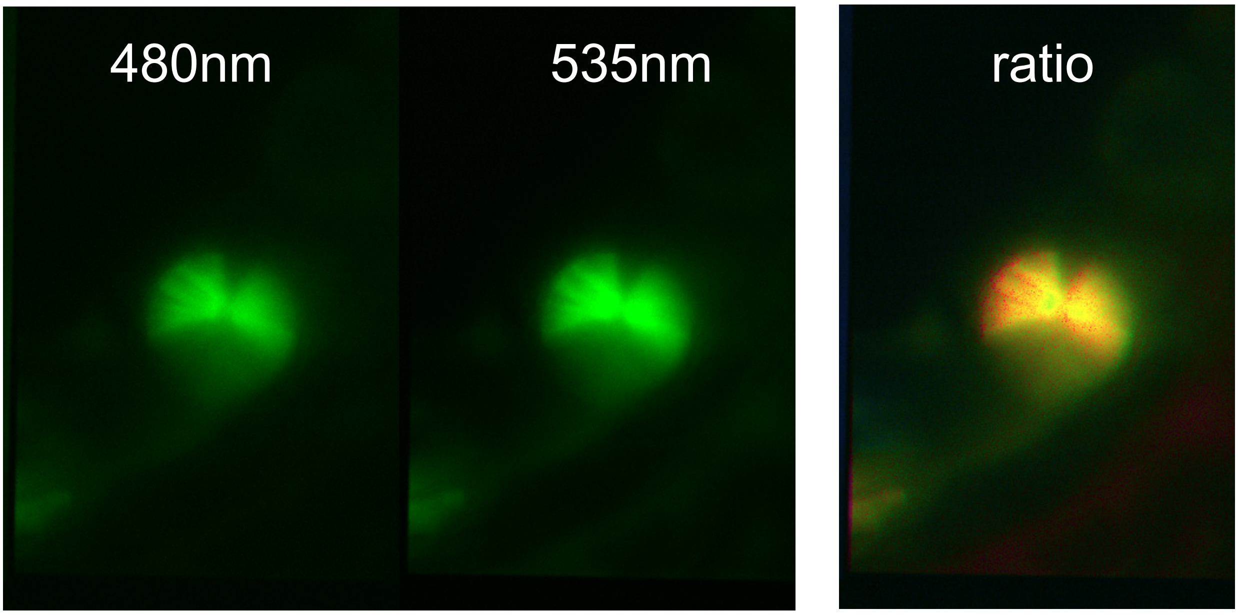

Fig. S1. Fluorescence images of C. elegans pharynx obtained simultaneously at both emission wavelengths, and ratio image F535/F480. Other details as indicated in Methods.

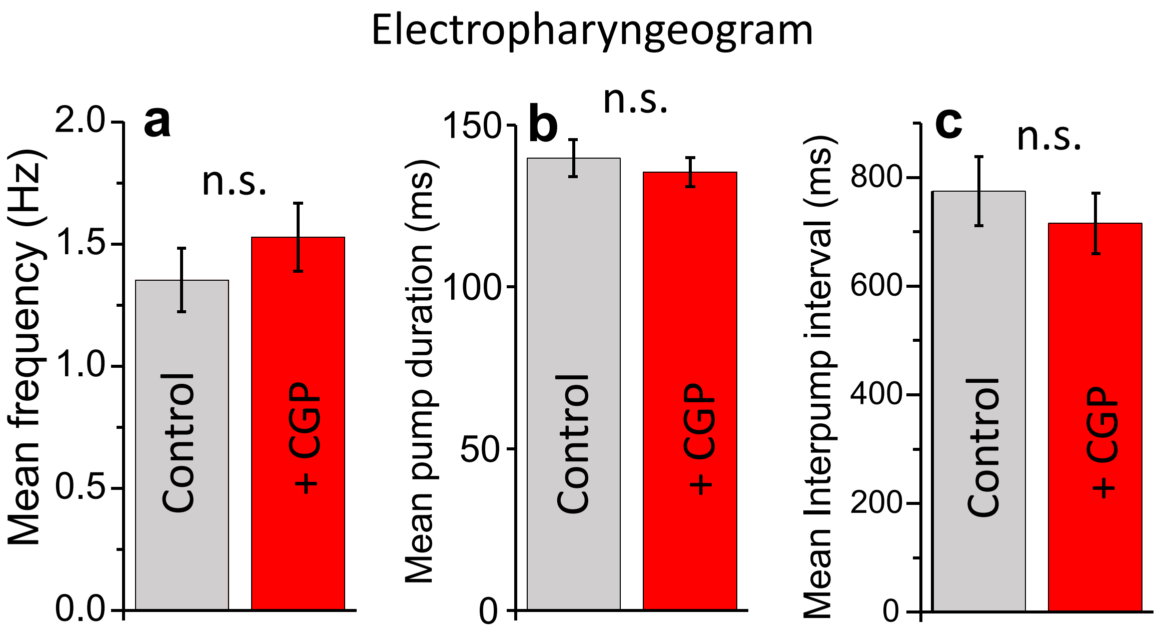

Fig. S2. Effect of CGP37157 on the electropharyngeogram. Panels a, b and c show the effect of 50µM CGP37157 on several parameters of the electropharyngeogram: mean frequency, mean pump duration and mean interpump interval, obtained from the analysis of data from 67 worms of each kind. There were no significant differences, ANOVA test. Other details as indicated in Methods.

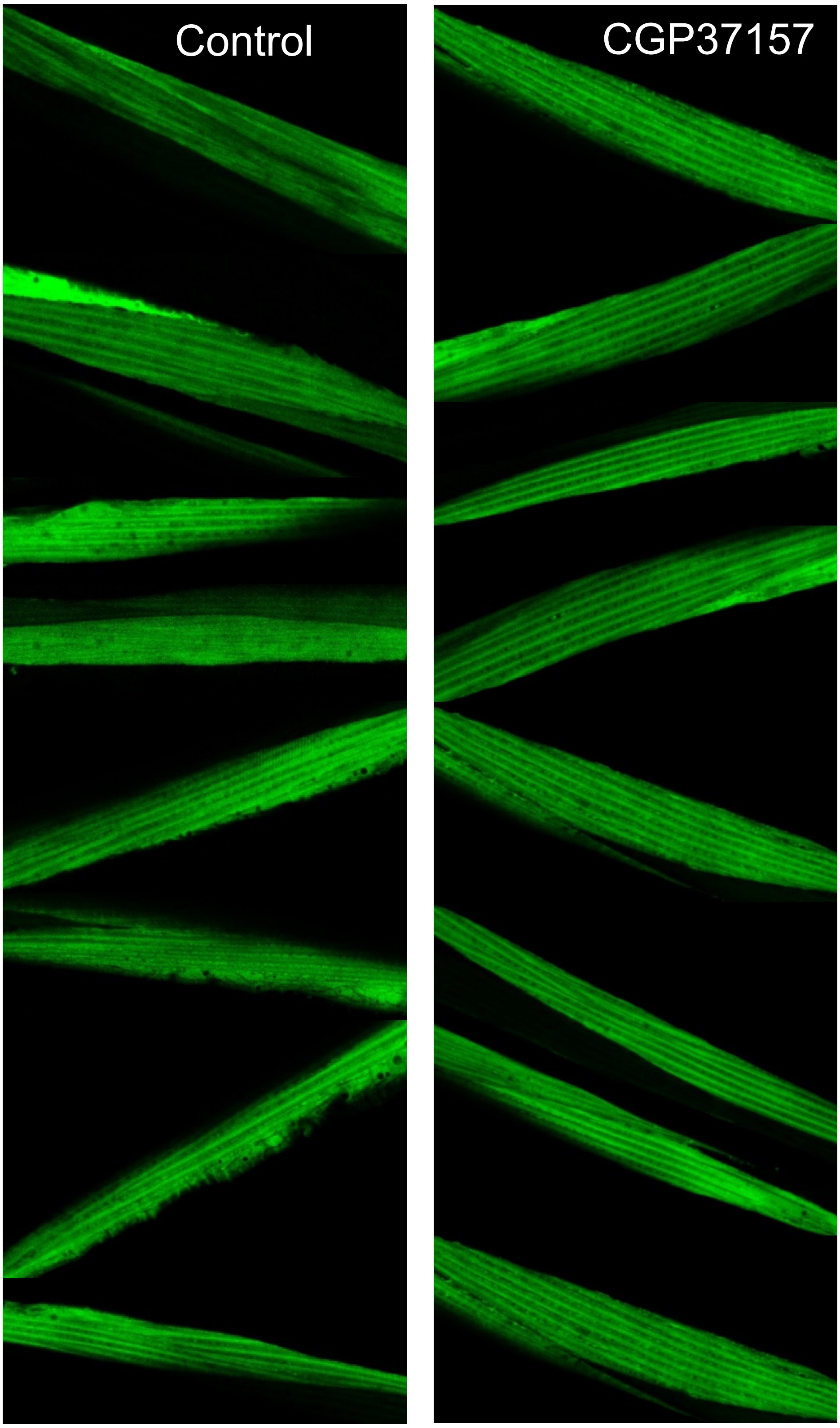

Fig. S3. Effect of CGP37157 on the sarcomeric structure in *C. elegans* body wall muscle at day 8 of adult life. The figure shows a series of typical images similar to those shown in Fig. 5 to show the sarcomeric structure at day 8 of adult life, both in control AQ2121 worms (expressing the YC2.1 fluorescent sensor in body-wall muscle cytosol) and in AQ2121 worms treated with CGP37157 50 μM since day 1.

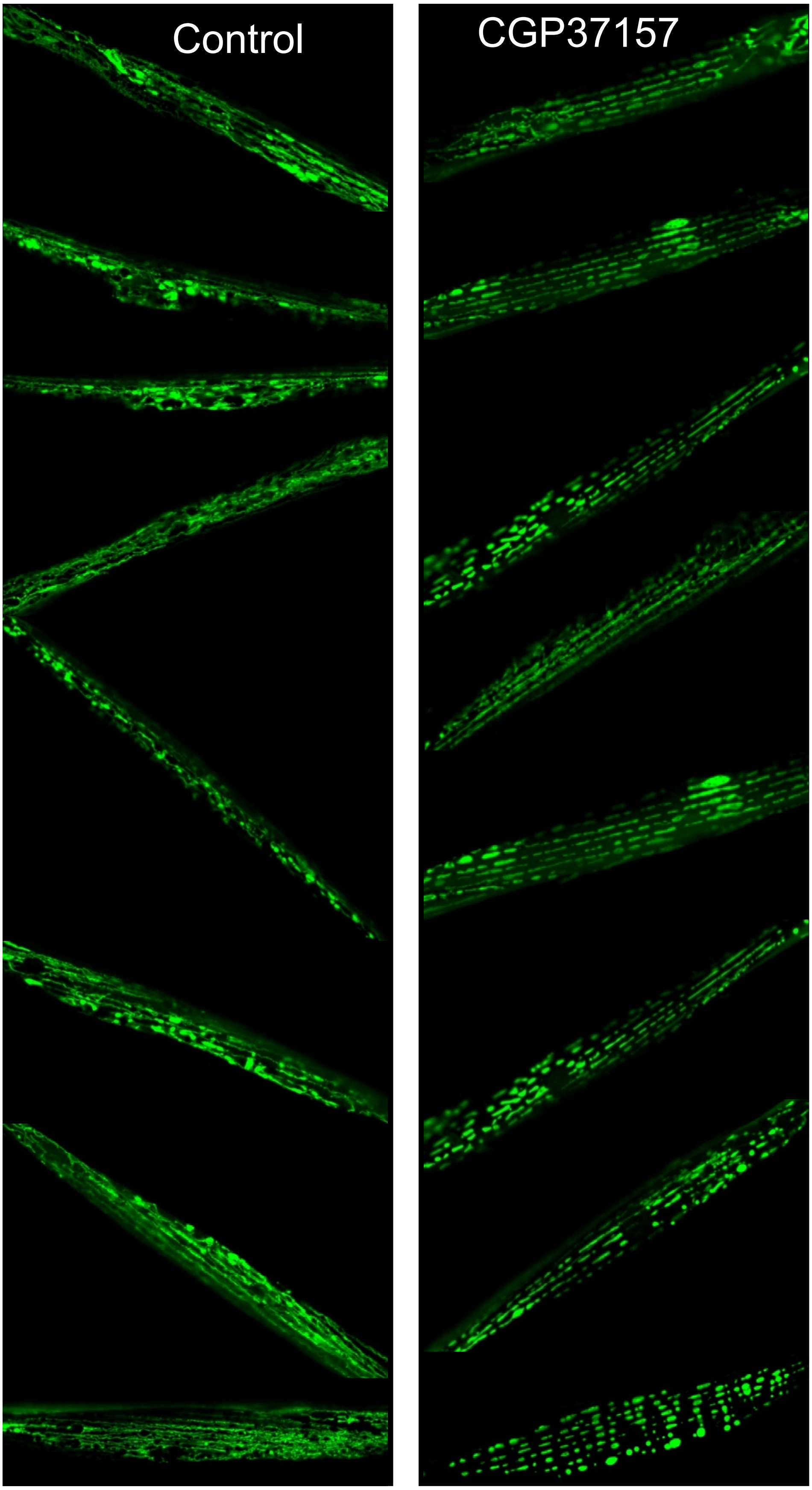

Fig. S4. Effect of CGP37157 on the mitochondrial structure in *C. elegans* body wall muscle at day 8 of adult life. The figure shows a series of typical images similar to those shown in Fig. 5 to show the sarcomeric structure at day 8 of adult life, both in control SJ4103 worms (expressing mitochondrial GFP in body-wall muscle cells) and in SJ4103 worms treated with CGP37157 50 μM since day 1.
