# Supplementary material for: The Mitochondrial Na+/Ca2+ Exchanger Inhibitor CGP37157 Preserves Muscle Structure and Function to Increase Lifespan and Healthspan in Caenorhabditis elegans
Source: Front Pharmacol. 2021 Jun 15;12:695687. doi: 10.3389/fphar.2021.695687 (PMC8241105; doi:10.3389/fphar.2021.695687)
Supplement: Supplementary file 4 [file DataSheet2.docx]

Supplementary Material

# Supplementary Tables

| **Table S1a. Treatment of *aak-2* mutant worms with CGP37157** | | | | | | |
| --- | --- | --- | --- | --- | --- | --- |
| Lifespan Drug (days) | N Drug | Lifespan Control (days) | N Control | % Lifespan increase | P value Drug vs Control | **Mean % lifespan increase** |
| 17.7 | 73/90 | 15.4 | 64/101 | 14.6 | <0,001 | **16.6±1.0** |
| 15.0 | 80/105 | 12.9 | 47/61 | 15.6 | <0.001 |  |
| 16.7 | 99/111 | 14.0 | 85/95 | 19.0 | <0.001 |  |
| **17.8** | **107/116** | **15.2** | **92/107** | **17.2** | **<0.001** |  |

| **Table S1b. Treatment of aak-1:*aak-2* mutant worms with CGP37157** | | | | | | |
| --- | --- | --- | --- | --- | --- | --- |
| Lifespan Drug (days) | N Drug | Lifespan Control (days) | N Control | % Lifespan increase | P value Drug vs Control | **Mean % lifespan increase** |
| **17.4** | **119/156** | **15.6** | **93/156** | **11.6** | **<0.001** | **9.6±1.3** |
| 16.1 | 104/125 | 14.4 | 101/112 | 11.4 | <0.001 |  |
| 15.9 | 109/121 | 15.0 | 102/121 | 6.13 | <0.001 |  |
| 14.8 | 108/119 | 13.6 | 86/109 | 9.2 | <0.001 |  |

| **Table S1c. Treatment of *rsks-1* mutant worms with CGP37157** | | | | | | |
| --- | --- | --- | --- | --- | --- | --- |
| Lifespan Drug (days) | N Drug | Lifespan Control (days) | N Control | % Lifespan increase | P value Drug vs Control | **Mean % lifespan increase** |
| 20.7 | 144/151 | 19.9 | 135/146 | 4.1 | 0.4 | **10.8±3.4** |
| 25.9 | 155/163 | 24.2 | 116/146 | 7.1 | <0.05 |  |
| 25.5 | 160/165 | 22.2 | 116/143 | 19.7 | <0.001 |  |
| **25.0** | **153/153** | **22.3** | **146/155** | **12.4** | **<0.001** |  |

**Table S1. Effect of CGP37157 on the lifespan of several *C. elegans* mutant worms.** The table shows the half-life of the worms incubated with the drug (Lifespan Drug) obtained from the Kaplan-Meier analysis, the number of worms in the drug-containing assay (N Drug, final/total), the half-life of the control worms (Lifespan Control), the number of worms in the control assay (N Control, final/total), the % increase in the half-life, the statistical significance of the difference between control and treated worms, obtained from the log-rank test, and the mean±s.e. increase in half-life for each mutant. In bold, series shown in the survival plots of Fig. 1.

| **Table S2a. Treatment of *sir-2.1* mutant worms with CGP37157** | | | | | | |
| --- | --- | --- | --- | --- | --- | --- |
| Lifespan Drug (days) | N Drug | Lifespan Control (days) | N Control | % Lifespan increase | P value Drug vs Control | **Mean % lifespan increase** |
| 24.4 | 96/109 | 17.9 | 88/101 | 36.0 | <0.001 | **39.3±2.4** |
| **30.0** | **91/107** | **21.7** | **81/90** | **37.8** | **<0.001** |  |
| 23.1 | 105/115 | 16.0 | 83/86 | 44.0 | <0.001 |  |

| **Table S2b. Treatment of *daf-16* mutant worms with CGP37157** | | | | | | |
| --- | --- | --- | --- | --- | --- | --- |
| Lifespan Drug (days) | N Drug | Lifespan Control (days) | N Control | % Lifespan increase | P value Drug vs Control | **Mean % lifespan increase** |
| 14.4 | 87/100 | 12.2 | 81/101 | 18.1 | <0.001 | **15.7±2.1** |
| **15.2** | **93/111** | **12.9** | **90/103** | **17.7** | **<0.001** |  |
| 16.7 | 82/98 | 15.0 | 82/92 | 11.5 | <0.001 |  |

**Table S2. Effect of CGP37157 on the lifespan of several *C. elegans* mutant worms.** In bold, series shown in the survival plots of Fig. 1. Other details as in Table S1.

**Table S3a. Effect of CGP37157 on mRNA expression.** Differential transcriptomic analysis made with the DESeq2 algorithm using the SARTools software. Only genes having a differential expression with p<0.05 have been included.

**Table S3b. Effect of CGP37157 on mRNA expression.** Differential transcriptomic analysis made with the EdgeR algorithm using the SARTools software. Only genes having a differential expression with p<0.05 have been included.
